# Improved cultivation and metagenomics as new tools for bioprospecting in cold environments

**DOI:** 10.1007/s00792-014-0704-3

**Published:** 2014-11-16

**Authors:** Jan Kjølhede Vester, Mikkel Andreas Glaring, Peter Stougaard

**Affiliations:** Department of Plant and Environmental Sciences, University of Copenhagen, Thorvaldsensvej 40, 1871 Frederiksberg C, Denmark

**Keywords:** Bioprospecting, Cultivation, Metagenome, Cold-active, Functional metagenomics, Multiple displacement amplification

## Abstract

Only a small minority of microorganisms from an environmental sample can be cultured in the laboratory leaving the enormous bioprospecting potential of the uncultured diversity unexplored. This resource can be accessed by improved cultivation methods in which the natural environment is brought into the laboratory or through metagenomic approaches where culture-independent DNA sequence information can be combined with functional screening. The coupling of these two approaches circumvents the need for pure, cultured isolates and can be used to generate targeted information on communities enriched for specific activities or properties. Bioprospecting in extreme environments is often associated with additional challenges such as low biomass, slow cell growth, complex sample matrices, restricted access, and problematic in situ analyses. In addition, the choice of vector system and expression host may be limited as few hosts are available for expression of genes with extremophilic properties. This review summarizes the methods developed for improved cultivation as well as the metagenomic approaches for bioprospecting with focus on the challenges faced by bioprospecting in cold environments.

## Introduction

Bioprospecting is the discovery and commercialization of new products such as enzymes and bioactive compounds based on biological resources. Generally speaking, bioprospecting of microorganisms can be conducted by culture-dependent or metagenomic approaches. The culture-dependent approaches are based on cultivation of natural isolates that can be screened for activities of interest, e.g., enzyme activities, antimicrobial activities or antibiotic resistances. However, only a small minority of microorganisms from an environmental sample can be cultured by standard techniques––often on conventional cultivation media in Petri dishes––and typically, less than 1 % of the total cell counts can be cultured in the laboratory (Amann et al. 1995). This discrepancy between the total number of bacteria from an environmental sample and the viable plate count has been termed “The Great Plate Count Anomaly” (Staley and Konopka 1985). The uncultivability of bacteria can be due to many factors, including lack of specific nutrients, oxygen level, temperature, pH and osmotic conditions as well as missing growth factors, possibly produced by other organisms in the community (Vartoukian et al. 2010). Culturing will always favor the organisms that are best at adapting to the conditions applied in the laboratory, and these organisms are not necessarily the most dominant or ecologically important organisms in the environment. Insights into the uncultured fraction have been fueled by sequencing of the 16S rRNA genes of bacteria from environmental samples, and this in turn has resulted in the identification of numerous new bacterial phyla, of which very few are represented by cultivated strains (Achtman and Wagner 2008; Stewart 2012).

Several methods that allow exploitation of the vast majority of microorganisms that are difficult to cultivate in the laboratory have been described. The methods can basically be divided into (1) improved cultivation techniques or (2) metagenomic methods. The improved cultivation techniques can be used to establish pure cultures or complex communities available for culture-dependent screenings or metagenomic approaches. The metagenomic methods are either based on sequencing of metagenomes and bioinformatic analysis or functional expression of metagenomic libraries to identify genes or gene clusters of interest.

Bioprospecting in extreme environments with non-standard conditions, such as low temperature, is met with additional challenges. Conventional cultivation may be difficult because of insufficient knowledge on media requirements or very prolonged incubation time, enrichment in situ may be problematic if the environment is located in remote areas, metagenomic analyses may be hampered by low amounts of environmental DNA, and heterologous expression of enzymes and other bioactive molecules may be challenging since most host organisms are not developed for extreme conditions. This review will present the methods developed for improved cultivation as well as the metagenomic approaches for bioprospecting with focus on the challenges faced by bioprospecting in cold environments. The methods that have been developed to deal with these problems will be summarized and possible routes to future improvements will be highlighted.

## Cultivation-based bioprospecting

A number of techniques have been developed for improved cultivation of natural microorganisms in the laboratory or in situ in the environment. Although only a few of these methods have been used specifically to bring cold-adapted microorganisms into culture, they all have the potential to increase the output of cultivation-based bioprospecting projects in cold environments. It is important, however, to keep in mind that cold-adapted microorganisms are faced with several challenges imposed by the low temperature. These include increased water viscosity, decreased diffusion rates, and not least reduced biochemical reaction rates (Feller 2013). For most biological systems, the activity of a mesophilic enzyme will be 16–80 times lower at 0 °C than at 37 °C (Georlette et al. 2004). In general, cold-active enzymes maintain a high biochemical reaction rate at low temperature by having a more flexible structure, resulting in a lower substrate affinity and increased heat lability (Feller and Gerday 2003; Feller 2013). Therefore, when designing experiments, care should be taken not to expose enzymes or organisms to elevated temperatures, as this might lead to irreversible inactivation of enzymes.

If communities or pure cultures can be established under laboratory settings, they will in many cases be suitable for bioprospecting efforts. Most techniques have been developed for traditional laboratory conditions, e.g., 20 °C, 1 atm., pH 7, etc., and are often not directly applicable to organisms originating from extreme environments. However, since most laboratories are equipped with facilities that allow incubation at low temperatures, screening of cultivated cold-adapted organisms can be carried out simply using existing assays at low temperature. A classic example is plate-based screening using chromogenic substrates that develop detectable colors upon activity (Fathallh et al. 2012), and by combining a mixture of different insoluble chromogenic substrates, multiple activities can be detected by various color combinations in single plate experiments (Ten et al. 2004, 2005). Subsequent identification and cloning of genes of interest have been aided by the emergence of genomics and access to affordable whole genome sequencing of bacterial isolates.

**Fig. 1:**
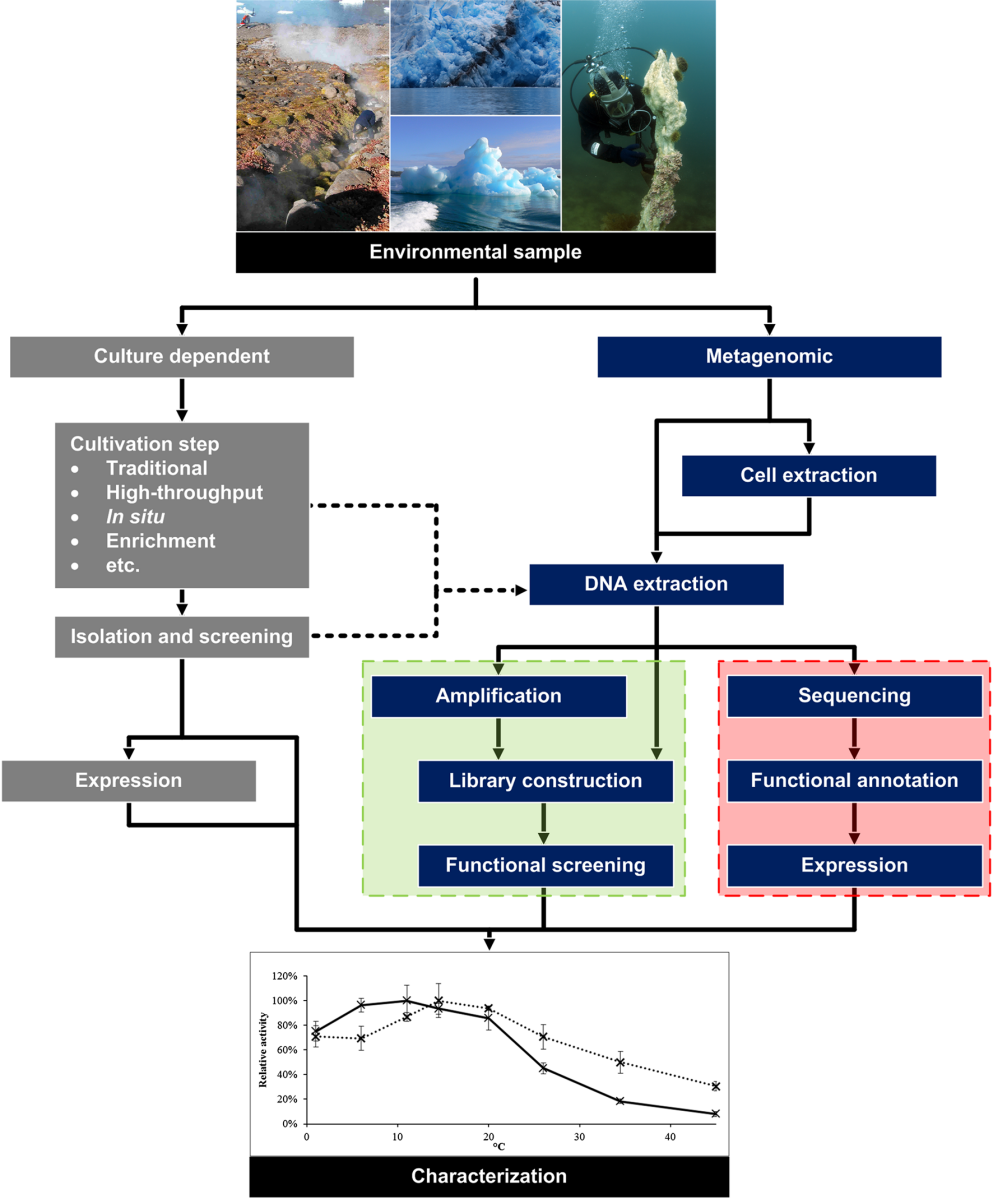
Simplified and general overview of culture-dependent and metagenomic methods for bioprospecting. In principle any environmental sample can be used. Culture-dependent methods (*gray boxes*) include a culturing step ranging from traditional culturing to various combinations of improved culturing techniques, leading to isolation and screening of natural isolates. Metagenomic methods (*blue boxes*) rely on DNA extraction, which can be either direct or indirect. Extracted DNA can be used for either sequence-based (*red*) or functional (*green*) screening approaches, where the latter might require amplification. Identified activities from natural isolates or recombinantly expressed proteins are then characterized. Links between culture-dependent and metagenomic methods include using DNA from enrichment cultures or natural isolates (*dotted lines*). See text for details. Inspired by Akondi and Lakshmi (2013)

The cultivation-dependent method of bioprospecting can involve several steps, ranging from traditional culturing to the various versions of optimized culturing conditions discussed below (Fig. 1). The subsequent isolation, screening and purification of microorganisms are typically done by plating on Petri dishes to obtain pure cultures for further analysis. Although improved cultivation methods may increase the proportion of cultivable microorganisms, many of these organisms will not grow as pure cultures in the laboratory. In addition, the specific gene or gene cluster responsible for the trait of interest often has to be identified to facilitate more efficient expression and characterization than possible with the natural isolate. Organisms solely capable of growing as microcolonies or in mixed communities can be analyzed by metagenomic approaches, which may also be applied directly to environmental samples. A useful coupling of culture-dependent and metagenomic methods is to use genomic DNA (gDNA) or cDNA from enrichment cultures or pure natural isolates for metagenomic methods (Hobel et al. 2004).

### Methods for improving cultivation

**Fig. 2:**
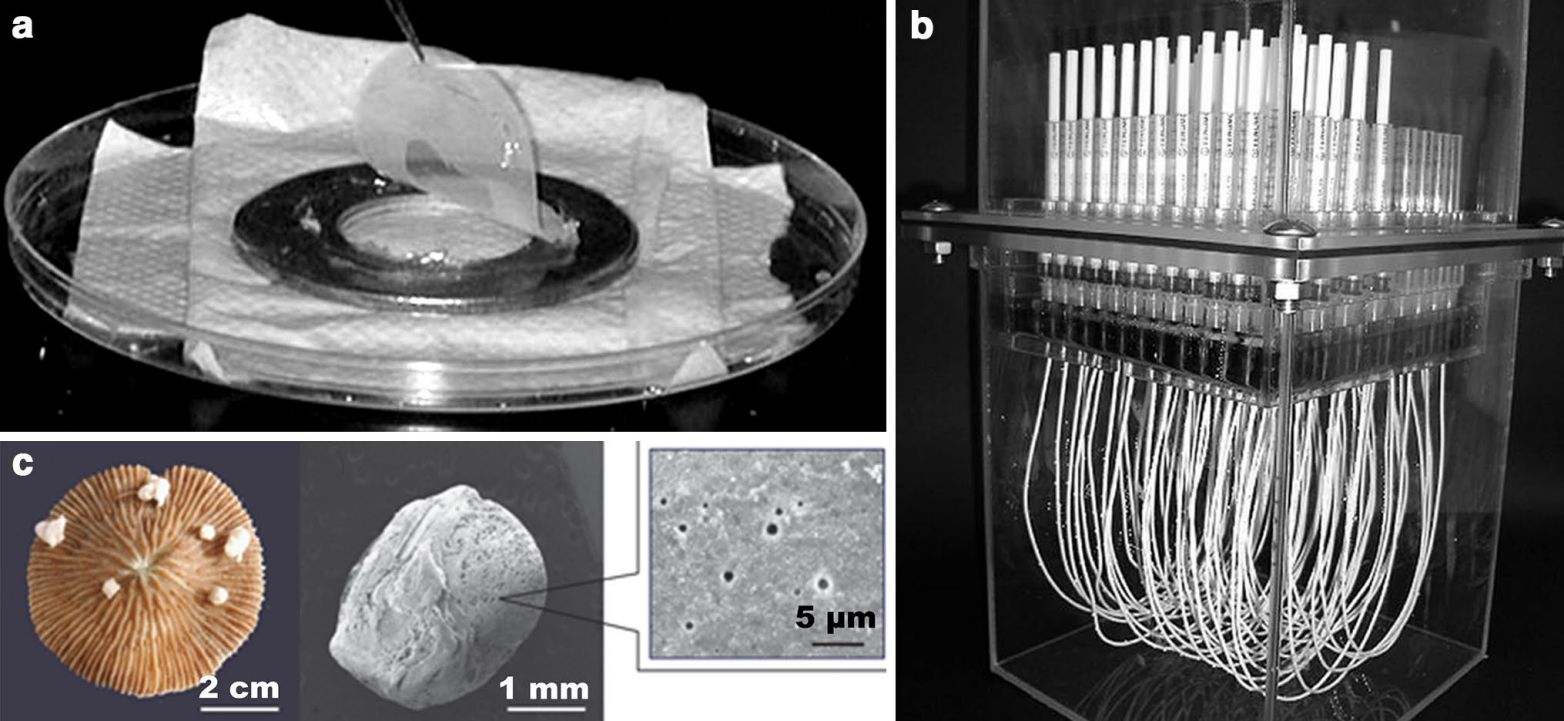
Cultivation techniques. Diffusion chamber (**a**), hollow-fiber membrane chambers (**b**), and encapsulated gel microdroplets (**c**). Modified from Kaeberlein et al. (2002, ©The American Association for the Advancement of Science), Aoi et al. (2009, ©American Society for Microbiology) and Ben-Dov et al. (2009, ©John Wiley and Sons), respectively

To bring more microorganisms into culture, it is reasonable to try to mimic the natural environment in the culturing conditions, e.g., by simulating temperature, pH, oxygen level or light, and using water or materials taken from the environment. An alternative taking this one step further is to bring the natural environment into the laboratory and use this for cultivation of microorganisms by keeping them separated from the environment in situ. Different methods have been developed using this approach, the three most prominent being; diffusion chambers, gel microdroplets, and hollow-fiber membrane chambers (Fig. 2). All of the techniques discussed below could potentially be applied to cold environments without further adjustments, although the various in situ incubation methods could prove difficult to apply for some extreme environments. Remote locations or hostile environments could restrict access as well as require improved performance of materials. In addition, limited knowledge on specific environments could make attempts at optimizing media composition and growth conditions difficult.

### Diffusion chamber and isolation chip (iChip)

In the diffusion chamber developed by Kaeberlein et al. (2002) (Fig. 2a), microorganisms are separated from their environment, embedded in agar and placed in a chamber closed by two permeable membranes. These membranes allow for diffusion of chemical components, and by placing the chamber in an aquarium simulating the natural environment, these components can pass through the membranes and stimulate growth of the separated cells in the chamber. The communities formed in the chambers can then be analyzed directly and purified by diluting into new chambers or by transferring to Petri dishes. Using fresh water sediments to inoculate diffusion chambers and placing them on the sediments for 4 weeks, Bollmann et al. (2007) were able to cultivate bacteria from rarely cultivated groups, and they found that continuous cultivation in diffusion chambers enabled growth of new bacteria in laboratory settings. A high-throughput version of the diffusion chamber, the isolation chip (iChip) developed by Nichols et al. (2010), consists of hundreds of miniature diffusion chambers, each of which can be inoculated with single cells to establish monospecific cultures. The iChip was tested on seawater and soil samples, and both total cell numbers as well as cultured diversity were increased compared to traditional culturing in Petri dishes.

### Hollow-fiber membrane chambers

The hollow-fiber membrane chambers developed by Aoi et al. (2009) take a similar approach by utilizing fibers that allow for exchange of metabolites with the environment. These fibers can be inoculated with microorganisms and placed in natural or engineered environments (Fig. 2b). The system was tested using samples from tidal sediment, activated sludge, and a laboratory bioreactor and led to an increase in diversity as well as in the number of novel cultivable species compared to culturing in Petri dishes (Aoi et al. 2009).

### Gel microdroplets and I-tip

Zengler et al. (2002) developed a high-throughput method for growing encapsulated microorganisms in gel microdroplets kept in a continuous fed-batch system with a low nutrient flux to simulate environmental conditions. The pores in the gel microdroplets allow for exchange of metabolites between organisms captured in separate droplets. Millions of microdroplets can be present in each growth column, and each growth column can be placed in specific media and growth conditions. Gel microdroplets with microcolonies are then identified by flow cytometry. The system was tested using seawater samples, and the highest diversity was obtained when filtered seawater was used as growth medium compared to seawater with nutrients added, suggesting that most of the diversity is outcompeted by relatively few fast growing organisms in a rich environment (Zengler et al. 2002). A technique combining the advantageous in situ incubation of diffusion chambers and hollow-fiber membrane chambers with the small size of the gel microdroplets was later developed by encapsulating microorganisms in agar and then further encasing them in a polysulfonic polymeric membrane, which permits exchange of chemical components and allows more flexible in situ incubation by being small (Fig. 2c). By inoculating with microorganisms from coral mucus and placing the encased agar on corals for incubation, approximately 50 % of the cultured organisms represented novel ribotypes (Ben-Dov et al. 2009). Unfortunately, no attempts were made to cultivate the microorganisms outside the encased agar. A similar method, the I-tip, was recently developed based on standard laboratory equipment (e.g., micropipette tips). It was used to cultivate bacteria associated with a sponge and was superior to a standard agar-based cultivation with respect to cultured diversity (Jung et al. 2014).

### Dilution to extinction

In the dilution to extinction technique, natural samples are diluted to very low cell concentrations and cultured to establish clonal populations. Connon and Giovannoni (2002) developed a high-throughput version of this method, which allows for cell enumeration of small volumes with low cell densities, making it possible to run the incubations in microtiter plates. This technique was used to culture members of the SAR11 clade which, despite its ubiquity, had previously evaded cultivation (Rappe et al. 2002).

### Resuscitation-promoting factors (Rpf)

Resuscitation-promoting factors (Rpf) are involved in resuscitation of dormant organisms and thereby in increasing their cultivability. Rpf have been intensively studied in *Mycobacterium tuberculosis* (Mukamolova et al. 1998; Kana et al. 2008) and this knowledge was used to cultivate the otherwise uncultivable strain *Psychrobacter* sp. strain MSC33 on standard media supplemented with a short Rpf peptide (Nichols et al. 2008).

### Gelling agent

Agar is by far the most commonly used gelling agent for microbiology. However, other gelling agents have been shown to be superior to agar in supporting growth of diverse bacteria. Using gellan gum, a water-soluble polysaccharide produced by *Pseudomonas elodea*, instead of agar, Tamaki et al. (2009) demonstrated that gellan gum not only supported much faster growth of bacteria from a freshwater lake sediment compared to agar, but also supported growth of more novel isolates than agar.

### Incubation time

There are several examples of increased cultivability with increased incubation time. Rarely isolated phylogenetic groups of soil bacteria have been cultivated by prolonged incubation (up to 3 months) (Davis et al. 2005; Steven et al. 2007) and a general increase in the diversity of haloarchaeal groups was observed with increasing incubation time (up to 12 weeks) (Burns et al. 2004). In another study, the cultured fraction of sea ice bacteria increased from 0.005 to 0.2 % if the incubation time was prolonged from 5 weeks to 3 months (Lanoil et al. 2009). Extended incubation time (more than 8 months) was found to significantly increase the diversity of cultured bacteria from a cold and alkaline environment, as well as the fraction of unknown genera and species (Vester et al. 2013). Also, many previously uncultured bacteria were discovered if multiple growth media were used (Joseph et al. 2003) and an increase in cultured diversity was observed if solid media were used rather than liquid (Vester et al. 2013).

### Sub-zero incubation

Cryophiles are extremely cold-adapted organisms capable of living in brine veins surrounding soil particles in permafrost soils or in ice brine veins at sub-zero temperatures. Such organisms can be cultured in media with high salt concentration supplemented with glycerol as shown for *Planococcus halocryophilus*, which was able to grow at temperatures as low as −15 °C (Mykytczuk et al. 2013).

## Metagenome-based bioprospecting

Since the culture-dependent methods only catch a very limited fraction of the total diversity of a given sample, metagenomic methods for bioprospecting can be highly valuable. Although these methods do not require cultivation, they may be restrained by the amount and quality of DNA obtainable from the environment or community of interest. The metagenomic approach can either be sequence based, involving high-throughput sequencing and bioinformatic analysis, or function based, aimed at functional expression of metagenomic libraries to identify genes or gene clusters of interest (Fig. 1).

### Sequence-based metagenomics

The sequence-based approach includes screening for genes by hybridization with labeled DNA probes or by PCR, both of which are based on sequences of known genes (Aakvik et al. 2011; Simon and Daniel 2011; Lee and Lee 2013). Since the price for direct high-throughput sequencing of metagenomic DNA has reached a level affordable for many laboratories (de Pascale et al. 2012; Hunter et al. 2012), this is now the standard technology for gene discovery (Ekkers et al. 2012). As outlined in Fig. 1, the sequence-based approach consists of three main steps: (1) the sequencing itself (2) bioinformatic analyses including functional annotation of genes, and finally (3) the heterologous expression of identified genes to document activity. For each of these steps, different opportunities exist and choices have to be made.

The various high-throughput sequencing platforms each have their advantages and disadvantages and the generated sequences subsequently go through a series of steps including quality control, filtering, assembly, and functional annotation of assembled sequences. Details of the sequencing platforms and sequence handling procedures have recently been reviewed by Loman et al. (2012) and Kim et al. (2013), respectively. It is important to note that functional annotation is generally based on sequence homology (Hunter et al. 2012; Kim et al. 2013) and since annotations are based on already known and characterized sequences, completely novel sequences are likely not to be annotated correctly or simply not annotated at all. To illustrate this, of six million proteins identified in a survey of fully sequenced bacterial genomes, the function of approximately 40 % was classified as unknown (Blaby-Haas and de Crecy-Lagard 2011).

The outcome of the sequencing and bioinformatics pipeline is typically a long list of genes of interest, which has to be investigated further to choose the best candidates for expression. Only 2.8 % of more than 20 million proteins in the UniProt database have had their existence confirmed at either the protein or transcript level (Temperton and Giovannoni 2012), indicating that a significant part of the annotated genes might not be expressed in the laboratory or at a too low level in Nature to be detected. Nevertheless, a prioritized list of expression targets can be made from analyses of properties of similar proteins, signal peptides, degree of novelty, etc., and the sequence information can be used for codon optimization and choice of expression organism.

### Function-based metagenomics

Functional metagenomics is the study of a metagenome by expression in a foreign host. This approach involves critical decisions regarding DNA fragment size, vectors and expression hosts (Ekkers et al. 2012). As for the sequence-based approach, the starting point is DNA (or cDNA). This has to be of good quality in terms of purity and fragment length. If the concentration of the purified DNA is too low for cloning, as is often the case for extreme environments, the DNA can be amplified by multiple displacement amplification (MDA) (Taupp et al. 2011).

### DNA isolation

Both gDNA and cDNA can be used for metagenomics and the DNA can be obtained from different sources including environmental samples (directly or indirectly isolated), enrichment cultures or pure isolates. Due to the difficulties in isolating mRNA from environmental samples, studies using cDNA are rare (Simon and Daniel 2011). For both enrichment cultures and pure isolates, isolation of mRNA is more feasible than for complex environmental samples. Also, if eukaryotic genes are of interest for a functional bioprospecting approach using a prokaryotic host for expression, cDNA is necessary to circumvent problems introduced by introns in gDNA (Aakvik et al. 2011). Direct extraction of DNA from environmental samples involves the in situ lysis of cells within the sample matrix and DNA extraction, whereas indirect sampling involves the isolation of cells from the sample matrix before ex situ lysis. The result from direct extraction is typically higher yield but more fragmented DNA, whereas indirect extraction yields are lower but with larger DNA fragments. Which method to choose depends on the sample matrix as well as the intended uses. Direct extraction can result in co-extraction of inhibitory substances such as humic acids from soil or the matrix may itself adsorb the DNA. Unwanted DNA such as eukaryotic or extracellular DNA can also constitute a significant part of the extracted DNA (Kakirde et al. 2010a; Aakvik et al. 2011; Williamson et al. 2011; Akondi and Lakshmi 2013; Lee and Lee 2013). These problems can be circumvented by indirect extraction. This is time consuming and biased, as it is unlikely that all types of cells separate from the sample matrix or remain intact during separation, with the result that the representativeness of the DNA will be affected (Ekkers et al. 2012). A study comparing direct and indirect DNA extraction from soil communities revealed that although the yield and quality of DNA were significantly different between the two methods, functional diversity was similar, probably due to functional redundancy in the community (Delmont et al. 2011). Bias is also introduced by the choice of lysis and DNA extraction method and it is likely that an accurate representation of the community will never be achieved. In general, it is advisable to apply multiple extraction methods, and when working with environments with low biomass, as is the case for many extreme environments, DNA yields are often low (Aakvik et al. 2011; Vester et al. 2014a, b). To obtain sufficient amounts of DNA for further analyses, MDA with the φ29 DNA polymerase can be applied (Spits et al. 2006). MDA is, however, heavily biased and will not generate quantitative results (Yilmaz et al. 2010; Vester et al. 2014b) and there is a risk of introducing point mutations (Aakvik et al. 2011).

DNA from more defined sources than environmental samples can also be used for bioprospecting. Several cold-active enzymes have been identified by screening genome libraries from single isolates with desirable properties, including a β-glucosidase from *Paenibacillus xylanilyticus* KJ-03 (Park et al. 2013), a β-d-galactosidase from *Paracoccus* sp. 32d (Wierzbicka-Wos et al. 2011) and an esterase from *Psychrobacter pacificensis* (Wu et al. 2013).

Enrichment cultures can be used to select for microorganisms with specific desirable traits. The relative fraction of DNA most likely to contain genes of interest is increased by reducing the overall complexity of the community (Cowan et al. 2005; Taupp et al. 2011; Ekkers et al. 2012). Among other studies, this approach has been used to enrich organisms capable of utilizing chitin as a carbon source at alkaline pH (Kielak et al. 2013) and identifying cellulolytic organisms (Grant et al. 2004). Although most microorganisms cannot be cultivated, more are able to grow in the mixed community of an enrichment culture than as pure isolates and using DNA extracted from these cultures will, therefore, yield different results than those obtained from culture-dependent screenings from the same enrichment culture (Entcheva et al. 2001; Voget et al. 2003). An alternative approach to enrichment is to use stable isotope probing (SIP) where a labeled (^13^C or ^15^N) substrate is added and utilized by metabolically active organisms that incorporate the heavier atoms into their DNA, which can subsequently be separated by density gradient centrifugation (Kakirde et al. 2010a; Ekkers et al. 2012).

### Vectors

The length of the fragments used for cloning depends on the type of screening library. There are three main types of functional expression libraries: plasmid (<15 kb), fosmid (25–35 kb)/cosmid (25–40 kb), and bacterial artificial chromosomes (BACs, 100–200 kb) (Ekkers et al. 2012). Plasmids are typically easy to handle and are suitable for single gene products such as most enzymes, and transformation efficiencies are high. Due to the small insert size, they are not useful when whole operons need to be detected (Taupp et al. 2011) and endogenous promoters might not be included. Using a plasmid with two promoters flanking the multiple cloning site may facilitate gene expression independently of insert orientation and endogenous promoter sequences (Lammle et al. 2007). For larger fragments, cosmids, fosmids, or BACs are used. These larger fragments and vectors are more difficult to handle, but offer the possibility of including entire operons and gene clusters as well as achieving a better sequence coverage with fewer clones. The commercially available CopyControl kit from Epicentre Biotechnologies can be used to generate fosmid libraries with ~40 kb inserts with a high cloning efficiency and stability in *Escherichia coli*, and is a frequently used method for expression libraries of metagenomic DNA (Table 1) (Lee and Lee 2013; Cheng et al. 2014). Since the hit rate in functional screening is typically very low (<2 out of 10,000 clones screened), high cloning efficiency and high-throughput screening are crucial for successful identification of activities (Akondi and Lakshmi 2013; Lee and Lee 2013). Shuttle vectors carrying multiple origins of replication can be used to move the library between different expression hosts, such as Gram-negative and Gram-positive bacteria. This is particularly useful for libraries made from environments with mixed communities.

**Table 1:** Cold-active enzymes identified by functional metagenomics

Enzyme	Host/vector	Positive/total clones	Screening technique	*T*_opt_	pH_opt_	Origin of sample	References
Lipase	*E. coli*/fosmid	70/>7,000	Agar based	35	N.A.	Baltic sea sediment	Hardeman and Sjoling (2007)
Lipase	*E. coli*/cosmid	N.A.	Agar based	30	7	Oil-contaminated soil (Northern Germany)	Elend et al. (2007)
Lipase	*E. coli*/fosmid	1/8,823	Agar based	25	8	Deep-sea sediment (Papua New Guinea)	Jeon et al. (2009a)
Lipase	*E. coli*/fosmid	1/6,000	Agar based	30	8	Intertidal sediment (Korea)	Kim et al. (2009)
Lipase	*E. coli*/plasmid	2/N.A.	Agar based	20	7–9	Soil from different altitude of Taishan (China)	Wei et al. (2009)
Lipase	*E. coli*/fosmid	1/2,400	Agar based	35	8	Mangrove sediment (Brazil)	Couto et al. (2010)
Lipase	*E. coli/fosmid*	1/386,400	Agar based	25	8	Tidal sediment (Korea)	Lee et al. (2012)
Lipase	*E. coli/fosmid*	6/81,100	Agar based	30–35	7.5–8.5	Deep-sea sediment	Jeon et al. (2011)
Esterase	*E. coli*/fosmid	1/N.A.	Agar based	50–55	10–11	Deep-sea sediment (Papua New Guiney)	Park et al. (2007)
Esterase	*E. coli*/fosmid	3/100,000	Agar based	40	9.0	Antarctic desert soil	Heath et al. (2009)
Esterase	*E. coli*/fosmid	6/60,132	Agar based	30	8	Arctic seashore sediment	Jeon et al. (2009b)
Esterase	*E. coli/fosmid*	1/N.A.	Agar based	35	7.5	Arctic intertidal sediment	Fu et al. (2013)
Esterase	*E. coli/fosmid*	3/100,000	Agar based	20	11	Antarctic desert soil	Hu et al. (2012)
Esterase	*E. coli/fosmid*	1/31,872	Agar based	35	8.5	Swamp sediment (Korea)	Seo et al. (2014)
Esterase	*E. coli/fosmid*	121/60,000	Agar based	20–30	9	Arctic soil	Yu et al. (2011)
Esterase	*E. coli/phage*	95/274,000	Agar based	15–40	8–10	Oil-contaminated seawater	Tchigvintsev et al. (2014)
Phthalate Esters Hydrolase	*E. coli/fosmid*	N.A./100,000	Agar based	10	7.5	Wastewater treatment plant (China)	Jiao et al. (2013)
Amylase	*E. coli*/cosmid	1/350,000	Agar based	40	6.5	Soil (Himalaya)	Sharma et al. (2010)
Amylase	*E. coli*/BAC	2/2,843	Agar based	15	8–9	Ikaite columns (Greenland)	Vester et al. (2014a)
Cellulase	*E. coli*/BAC	11/10,000	Agar based	10–50	6–9	Antarctic soil	Berlemont et al. (2009)
Cellulase	*E. coli*/plasmid	1/8,500	Agar based	28	4.5	Cold desert (Himalaya)	Bhat et al. (2013)
Cellulase	*E. coli/plasmid*	1/40,000	Agar based	40	7	Brown alga associated microorganism (France)	Martin et al. (2014)
β-Galactosidase	*E. coli/*plasmid	3/1,200	Agar based	38	7	Topsoil of oil field (China)	Wang et al. (2010)
β-Galactosidase	*E. coli*/BAC	2/2,843	Agar based	37	6–7	Ikaite columns (Greenland)	Vester et al. (2014b)
β-Galactosidase	*E. coli*/plasmid	1/1,100	Agar based	40	6.5	Baltic sea water	Wierzbicka-Wos et al. (2013)
Xylanase	*E. coli*/phagemid	1/5,000,000	Agar based	20	6–7	Waste lagoon of dairy farm (California)	Lee et al. (2006)
Chitinase	*E. coli/fosmid*	1/29,000	Agar based	30	N.A.	Genomes from Antarctic soil (1) and Arctic sea (9) bacteria	Kim et al. (2014)
DNA polymerase I	*E. coli*/plasmid and fosmid	15/23,000 and 1/4,000	Growth assay	N.A.	N.A.	Glacial ice (Germany)	Simon et al. (2009)

Adapted and updated from Cavicchioli et al. (2011, ©John Wiley and Sons)

*N.A.* not applicable or not available

### Expression hosts

The choice of expression host will influence the design and result of functional expression on all levels. *E. coli* is by far the most commonly used host due to the substantial genetic toolbox available (Aakvik et al. 2011), but methods for using other organisms have been developed. Functional expression faces the same challenges associated with heterologous expression: codon usage, improper promoter recognition, missing initiation factors, protein misfolding, missing co-factors, breakdown of product, improper secretion of product, toxicity of product or intermediates, and formation of inclusion bodies (Ekkers et al. 2012). Since the origin of metagenomic DNA is unknown, it is impossible to predict the effect of these potential problems, as they may vary from gene to gene and may depend on the expression host. Gabor et al. (2004) estimated that 40 % of the genes from 32 different bacterial and archaeal genomes contained expression signals that would be recognized in *E. coli*. However, the actual fraction of genes that can be successfully expressed in *E. coli* is probably significantly lower, as missing co-factors, chaperones, secretion, etc., was not considered. Not surprisingly, heterologous expression in *E. coli* is more efficient for closely related organisms (Warren et al. 2008). *E. coli* has been engineered to increase the expression of heterologous genes by improving the recognition of ribosomal binding sites, expressing more chaperones, and/or enhancing secretion (Aakvik et al. 2011; Ekkers et al. 2012). An example of such an engineered host strain is the Arctic Express *E. coli* strain (Agilent Technologies), which has been optimized for low temperature expression by inclusion of chaperones from the psychrophilic bacterium *Oleispira antarctica*. This is beneficial since the native *E. coli* chaperones have highly reduced activity at low temperatures. Also, by engineering the EPI300 *E. coli* strain of the CopyControl kit from Epicentre to express two additional sigma factors from *Clostridium* and *Streptomyces*, the hit rate in a functional screening was increased by 20–30 % (Liebl et al. 2014). Alternatively, the psychrophilic Gram-negative bacterium *Pseudoalteromonas haloplanktis* TAC125 has been established as a system for expression and secretion of recombinant proteins (Cusano et al. 2006). To deal with improper secretion, Li et al. (2007) developed a vector system that enables forced cell lysis inducible by UV radiation to avoid the use of costly, time-consuming, and possibly denaturing lysing agents.

If phylogenetic information on the metagenomic DNA is available, or if the proteins of interest are known to be highly abundant in a specific type of bacteria, a related host can be chosen to enhance the chance of successful expression. Alternatively, shuttle vectors that allow for library expression in multiple hosts can be used. The pCC1FOS vector for fosmid and BAC cloning, which normally only replicates in *E. coli*, has been modified so it can be transferred to other species by conjugation (Aakvik et al. 2009). Another broad-host-range vector is the pGNS-BAC vector, which features an inducible copy number and has been successfully used in six different *γ*-*Proteobacteria* (Kakirde et al. 2010b). The broad-host-range cosmid vector, pJWC, was used to establish a metagenomic library in six different *Proteobacteria*, including *α-*, *β-*, and *γ-Proteobacteria*, with very little overlap in expression (Craig et al. 2010), confirming the usefulness of multiple hosts.

The shuttle vectors mentioned above have been used in organisms from the same phylum as *E. coli* and may not be suitable for expression of genes from more distantly related organisms (e.g., Gram-positive bacteria). They can, however, be useful for transferring a library to a host better adapted to the screening conditions. As an example, *E. coli* is not an optimal host for screening for cold- and alkaline-active enzymes since it does not grow well at low temperatures and not at all at high pH, but a library can be established in *E. coli* and subsequently transferred to a related host adapted to these conditions to allow for direct assays at low temperature and high pH (Liebl et al. 2014). To deal with a similar problem, Angelov et al. (2009) developed a two-host fosmid system, which permits transfer of a library from *E. coli* to the thermophilic Gram-negative *Thermus thermophilus* and confirmed that the expression patterns between these two organisms were very different. BAC shuttle vectors that can replicate in both Gram-positive and Gram-negative bacteria have been developed (Hain et al. 2008; Ouyang et al. 2010), including the *E. coli*–*Bacillus subtilis* shuttle vector pHT01 from MoBiTech (Biver et al. 2013). Additional commercial shuttle vectors with increased transfer efficiency and flexibility are currently being developed (David Mead, Lucigen, personal communication), which could prove beneficial for increasing the expression rate in metagenomic libraries. Also, the pCC1FOS vector has been modified to allow transfer to *Mycobacterium* spp. (Ly et al. 2011), and McMahon et al. (2012) developed a shuttle vector for *E. coli* and *Streptomyces lividans* and an optimized *S. lividans* strain for expression. Recently, Cheng et al. (2014) created cosmid vectors for transfer of cloned metagenomic DNA by pentaparental conjugation to Gateway^®^ destination vectors that are able to replicate in hosts such as *Bacillus* and *Saccharomyces*.

### Screening

The challenges and limitations of the function-based approach highlight the necessity for a robust and high-throughput screening setup with low detection threshold to capture the few positive clones (Taupp et al. 2011). With function-based metagenomics, it is possible to perform heterologous complementation screenings where the metagenomic inserts complement a given trait in the host organism with the advantage that only positive clones will be able to grow. This approach was used to screen for cold-active DNA polymerase activities using an *E. coli* strain with a cold-sensitive lethal mutation in DNA polymerase I (Nagano et al. 1999) as a host for a metagenomic library from glacial ice (Simon et al. 2009). Clones with resistance to antibiotics or heavy metals can be selected for by including these substances in the growth medium (Kakirde et al. 2010a).

Another approach for functional screening is substrate-induced gene expression screening (SIGEX) where metagenomic DNA is cloned upstream of a promoter-less GFP. This allows for detection of promoters induced by the conditions applied after which, cells can be sorted using fluorescence-activated cell sorting (FACS). This method has been used to identify genes induced by aromatic-hydrocarbon compounds in a groundwater metagenome library in *E. coli* (Uchiyama et al. 2005). A similar approach is the product-induced gene expression screening (PIGEX) where a reporter strain with a product-sensitive promoter coupled to GFP is co-cultivated with a metagenomic library to facilitate detection of product formation by fluorescence. This has been used to identify amidase activities in *E. coli* carrying a metagenome library from activated sludge of a wastewater treatment facility (Uchiyama and Miyazaki 2010).

The functional approach has significant benefits, most importantly that it enables the detection of truly novel activities as no prior sequence information is needed. Furthermore, targeted activities are often directly available in a relevant production organism, making the road to optimized expression less troublesome than for the sequence-based approach. Among the main disadvantages are the rather labor-intensive setup and the necessity for high-throughput screening to maintain a reasonable hit rate (Aakvik et al. 2011; Simon and Daniel 2011; Taupp et al. 2011).

### Cold-active enzymes identified by functional metagenomics

Table 1 lists cold-active enzymes identified by functional metagenomics. It can be seen that bioprospecting for cold-active enzymes has been conducted in many different environments including sediments from both poles, the deep-sea, cold deserts, mountain soils, lakes, and glacial ice. The characteristic low hit rate as well as the preferred use of fosmid libraries and *E. coli* as host is also obvious. Lipases and esterases dominate the enzyme activities identified, most likely as a result of the easy detection of these activities. The results clearly demonstrate the need for further development of alternative hosts and assays to facilitate easy and efficient screening for additional enzyme activities, since cold-active enzymes have many useful applications.

## Applications of cold-active enzymes

Enzymes from psychrophiles are generally cold active and heat labile, which has three main advantages for biotechnological applications: (1) because of the higher specific activity, less (expensive) enzyme is required; (2) processes can run at the temperature of tap water or ambient temperature reducing the need for heating; and (3) their activity can be (selectively) inactivated by a moderate temperature increase (Feller 2013). Many industrial and biotechnological processes make use of cold-active enzymes or could benefit from their use, as the reduced temperature can be beneficial in multiple ways. Such processes may save energy and production costs, improve hygiene, maintain taste and other organoleptic properties, and reduce the risk of contamination. Cold-active enzymes are used in fine chemical synthesis, environmental biotechnology, biofuels and energy production, and in the food and feed, detergent, pharmaceutical, medical and textile industries (Cavicchioli et al. 2011). Specific examples include the use of cold-active β-galactosidases to hydrolyze lactose to generate lactose-free milk (Feller 2013), heat-labile α-amylases in the baking industry (Coronado et al. 2000) as well as heat-labile phosphatases and nucleases for molecular biology (Feller 2013). In laundry and dish-washing detergents, the use of cold-active enzymes (proteases, cellulases, lipases, and amylases) is especially promising, since they allow for environment-friendly low temperature washing (Horikoshi 1999; van der Maarel et al. 2002; Fujinami and Fujisawa 2010; Mojallali et al. 2013).

## Conclusions and perspectives

Approximately 75 % of the Earth’s biosphere is cold (<5 °C) (Huston 2008), and since microbial life is present practically everywhere, this constitutes an enormous reservoir for bioprospecting for cold-adapted activities of interest. Culture-dependent bioprospecting is an important and valuable tool in this regard, but since most microorganisms cannot be easily cultured, if at all, it is limited to covering only a fraction of the total biodiversity. The large uncultivable fraction of microorganisms can potentially be accessed by improved cultivation or through metagenomic approaches and these technologies each have their advantages and disadvantages. Culture-based methods result in organisms that are known to produce potentially novel, active enzymes but the re-discovery rate can be very high (Aakvik et al. 2011; Vester et al. 2014a, b). Sequence-based metagenomics often identifies a large number of genes encoding putative enzyme activities, but there is no guarantee that the genes can be expressed as active enzymes in available heterologous hosts and it also relies on previous sequence knowledge for identification. Function-based metagenomics may result in novel, functionally active enzymes, but the hit rate can be extremely low.

Bioprospecting at low temperature is faced with additional difficulties including low biomass and DNA availability, and the lack of cold-adapted expression hosts for functional metagenomics. Both academia and industry could benefit from a more diverse collection of host strains with various extremophilic properties, including adaptation to low temperature, to increase the likelihood of identifying specific activities of interest. Furthermore, the development of more versatile vectors as well as strain engineering should improve the low hit rate associated with functional metagenomics. The potential of the uncultured microbial diversity is enormous, and with the methods presently available and those currently being developed, this biological resource is becoming increasingly accessible.

## Abbreviations

BAC: Bacterial artificial chromosome
gDNA: Genomic DNA
iChip: Isolation chip
MDA: Multiple displacement amplification
PIGEX: Product-induced gene expression screening
Rpf: Resuscitation-promoting factors
SIGEX: Substrate-induced gene expression screening
SIP: Stable isotope probing
FACS: Fluorescence-activated cell sorting
